# Associated factors and outcomes of social activity restriction in patients with chronic obstructive pulmonary disease: a real-world longitudinal cohort study

**DOI:** 10.7189/jogh.16.04295

**Published:** 2026-10-23

**Authors:** Cong Liu, Zijing Zhou, Qing Song, Tao Li, Ping Zhang, Ling Lin, Yating Peng, Yuqin Zeng, Yan Chen, Shan Cai, Ping Chen

**Affiliations:** 1 Center of Respiratory Medicine, Xiangya Hospital, Central South University, Changsha, Hunan, China; 2 National Key Clinical Specialty, Branch of National Clinical Research Center for Respiratory Disease, Xiangya Hospital, Central South University, Changsha, Hunan, China; 3 Hunan Engineering Research Center for Intelligent Diagnosis and Treatment of Respiratory Disease, Changsha, Hunan, China; 4 National Clinical Research Center for Geriatric Disease (Xiangya Hospital), Central South University, FuRong Laboratory, Changsha, Hunan, China; 5FuRong Laboratory, Changsha 410078, Hunan, China; 6 Department of Pulmonary and Critical Care Medicine, the Second Xiangya Hospital, Central South University, Changsha, Hunan, China; 7 Research Unit of Respiratory Disease, Central South University, Changsha, Hunan, China; 8 Clinical Medical Research Center for Pulmonary and Critical Care Medicine in Changsha, Hunan, China; 9 Diagnosis and Treatment Center of Respiratory Disease in Hunan Province, Changsha, Hunan, China

**Keywords:** chronic obstructive pulmonary disease, social activity, acute exacerbation, mortality

## Abstract

**Background:**

Social activity restriction has emerged as a new public health challenge; the factors associated with social activity restriction and its outcomes in patients with chronic obstructive pulmonary disease (COPD) are not yet clear. In this study, we aimed to describe the clinical characteristics of socially restricted COPD patients and to evaluate the effects of social activity restriction on acute exacerbation and mortality.

**Methods:**

We included participants enrolled in the RealDTC study between December 2016 and August 2020. We collected data on demographics, degree of social activity restriction, lung function, Clinical COPD Questionnaire score, and mortality at four years. We used multivariable regression analyses to examine whether restricted social activity was associated with the risks of acute exacerbation and mortality.

**Results:**

We included 3,679 patients in total. Their mean age was 65.72 (standard deviation = 9.1) years, and 11.3% had restricted social activities. Older age, widowhood, GOLD grades 3–4, Global Initiative for Chronic Obstructive Lung Disease groups B and D, history of acute exacerbation, COPD Assessment Test score of ≥10, and Modified Medical Research Council score of ≥2 were independently associated with social activity restriction. By multivariable analysis, patients with restricted social activities had higher risks of future acute exacerbation (adjusted odds ratio (aOR) = 1.320; 95% confidence interval (CI) = 1.100–1.583, *P* = 0.003) and severe acute exacerbation (aOR = 1.299; 95% CI = 1.014–1.664, *P* = 0.038). Furthermore, restricted social activity was an independent risk factor for mortality (hazard ratio = 1.752, 95% CI = 1.298–2.364, *P* < 0.001).

**Conclusions:**

Restricted social activity was not uncommon among patients with COPD. Social activity restriction should be routinely assessed in COPD clinics as a simple marker to identify patients at increased risk of exacerbation and mortality. During future epidemics or pandemics, public health responses should be more flexible, risk-stratified, and patient-centred for individuals with chronic respiratory disease. Future interventional and policy studies are needed to determine whether systematic assessment of social activity restriction and targeted supportive programs can improve outcomes in socially restricted patients with COPD.

Chronic obstructive pulmonary disease (COPD) is a common and preventable chronic airway disease characterised by persistent airflow limitation [1]. In China, the prevalence of COPD has increased significantly compared with previous estimates (8.2% *vs.* 13.8%) [2,3]. COPD is expected to impose an even greater burden on public health with the accelerated ageing of the population in China [4]. Therefore, identifying preventable factors associated with COPD is particularly important.

Social isolation (lack of family contact, social network interaction, and community participation) affects public health and has emerged as a new public health challenge [5], which may be associated with reduced physical activity, progressive skeletal muscle deconditioning, exercise intolerance, and frailty, all of which are established predictors of exacerbation and mortality in COPD [6]. It may also coexist with depression, anxiety, loneliness, and reduced self-efficacy, which can impair medication adherence, smoking cessation, symptom monitoring, and participation in pulmonary rehabilitation [7,8]. These pathways suggest that restricted social activity may serve not only as a marker of disease severity but also as a potential contributor to adverse COPD outcomes.

In the general population, socially inactive individuals have greater hospitalisation needs and higher hospitalisation costs than socially active individuals [9]. Social inactivity has also been associated with an increased risk of all-cause mortality [10]. Patients with COPD often experience symptoms such as dyspnoea, cough, sputum production, and fatigue, which can lead to less interaction with friends and family and a reduced ability to participate in social activities [11,12]. Kochovska *et al.* [13] found that 37.8% of elderly men with COPD and/or vascular disease had restricted social activities due to dyspnoea. A cross-sectional study in China found that 25.7% (n/N = 37/148) of patients with COPD reported restricted social activities [14]. Iyer *et al.* [15] found that restricted social activity was a risk factor for severe acute exacerbation after following 50 patients with COPD for one year. However, a small number of large-sample studies describe factors associated with restricted social activity, and its impact on long-term prognosis remains unclear. Therefore, we used data from a real-world observational cohort study (RealDTC study) to describe the clinical characteristics of socially restricted patients with COPD and to evaluate their risks of acute exacerbation and mortality.

## METHODS

### Study participants

We included participants from the RealDTC study (registration number: ChiCTR-POC-17010431), a prospective real-world cohort study [16]. We included participants enrolled between December 2016 and August 2020. The diagnostic criteria for COPD were based on the Global Initiative for Chronic Obstructive Lung Disease (GOLD) 2017 document, with a post-bronchodilator forced expiratory volume in 1 second (FEV_1_)/forced vital capacity (FVC) ratio of <0.7 [1]. We excluded participants with an inability to communicate because of cognitive dysfunction; the presence of severe liver or kidney dysfunction, cardiac insufficiency, or malignant tumours; and the lack of key information, such as Clinical COPD Questionnaire (CCQ) data, acute exacerbation history, or mortality status. The Institutional Review Board of the Second Xiangya Hospital of Central South University approved our study (No. 2016076), and we conducted it in accordance with the Declaration of Helsinki. We obtained written informed consent from all participants.

### Data collection

We collected baseline data, including age, sex, marital status, education level, smoking status, lung function, COPD Assessment Test (CAT) score, modified Medical Research Council (mMRC) dyspnoea scale score, CCQ score, and history of acute exacerbation, at the time of enrollment. We collected information on moderate-to-severe acute exacerbations at the one-year follow-up, and mortality data at the four-year follow-up.

### Definition of variables

We defined non-smoking as a cumulative smoking exposure of fewer than 100 cigarettes, and smoking cessation as abstinence from smoking for more than six months. Following the GOLD 2017 document, we classified patients into four GOLD groups (A–D) based on the number of acute exacerbations in the previous year, CAT score, and mMRC score [1]. Based on post-bronchodilator FEV_1_ per cent predicted (FEV_1_%pred), we classified patients with FEV_1_%pred of ≥50% as GOLD grades 1–2, and those with FEV_1_%pred of <50% as GOLD grades 3–4. Moderate acute exacerbation was defined as worsening respiratory symptoms requiring oral corticosteroids and/or antibiotics, and severe acute exacerbation as worsening symptoms requiring an emergency department visit or hospital admission [1]. To evaluate the impact of the COVID-19 pandemic on restricted social activity, we divided patients into pre-pandemic and post-pandemic groups according to their enrolment date, with early 2020 used as the cutoff. Social activity restriction was assessed using item 10 of the CCQ, which records the degree of restriction in social activities resulting from COPD-related health status (*e.g.* talking, spending time with children, and visiting friends and relatives). The CCQ has demonstrated good reliability, responsiveness, and concurrent validity with established measures such as the St. George’s Respiratory Questionnaire and CAT [17]. Although the CCQ was not designed as a dedicated social isolation scale, its functional and social domains have been used in previous studies to evaluate activity limitation [18]. Accordingly, we used the CCQ social activity item to capture disease-related restriction of social participation rather than social isolation or loneliness *per se*. We considered a score of 0–1 on CCQ item 10 as no social activity restriction, whereas we defined a score of ≥2 as social activity restriction. We further categorised the degree of social activity restriction into three levels: 0–1 (none), 2–3 (moderate restriction), and 4–6 (severe restriction).

### Statistical analysis

The proportion of missing data was low (<5%) for all key variables. Given this low level of missingness, we conducted the primary analyses using complete-case analysis, including only patients with complete data for the exposure, outcomes, and covariates in each model. We used the Kolmogorov-Smirnov test to assess the distribution of variables. We presented continuous variables as mean (x̄) standard deviation (SD), whereas categorical variables as absolute numbers and percentages. We used Student’s *t*-test for comparisons of normally distributed continuous variables, and the Mann-Whitney U test for non-normally distributed variables. We evaluated multicollinearity among covariates in the multivariable models using the variance inflation factor, with all values below five indicating the absence of significant multicollinearity. We assessed the proportional hazards assumption for the Cox proportional hazards models using scaled Schoenfeld residuals and log-minus-log survival plots; when the assumption was not met, we used time-varying covariates or stratified Cox models. To assess the robustness of our findings and distinguish the effect of social activity restriction from bereavement, we performed several sensitivity analyses. First, we re-estimated the multivariable models with marital status forced into the models as a covariate. Second, we repeated the primary analyses after excluding widowed patients. We considered a two-sided *P*-value of <0.05 statistically significant.

We performed all statistical analyses using SPSS, version 26.0 (IBM Corp., Armonk, NY, USA).

## RESULTS

### Clinical characteristics of COPD patients with restricted social activity

We included 3,679 patients with COPD in total (**Figure 1**), with a mean age of 65.72 (SD = 9.1) years. The age distribution by decade is provided in Table S1 in the **Online Supplementary Document**, showing the proportions of patients in their 40s, 50s, 60s, 70s, 80s, and 90s. Of these patients, 84.6% were male, 58.9% had a high school education or above, 43.6% were current smokers, 54.1% were classified as GOLD grades 1–2, 37.4% belonged to GOLD group D, and 11.3% had restricted social activity. Compared with patients without restricted social activity, those with restricted social activity were older and had higher CAT, mMRC, and CCQ scores. They also had a higher proportion of widowed individuals, GOLD grades 3–4, GOLD group D, a history of acute exacerbation, CAT score of ≥10, and mMRC score of ≥2, as well as lower body mass index, FEV_1_, FEV_1_%pred, and FEV_1_/FVC. We found no significant differences between the two groups in sex, education level, or smoking status (**Table 1**).

**Figure 1:**
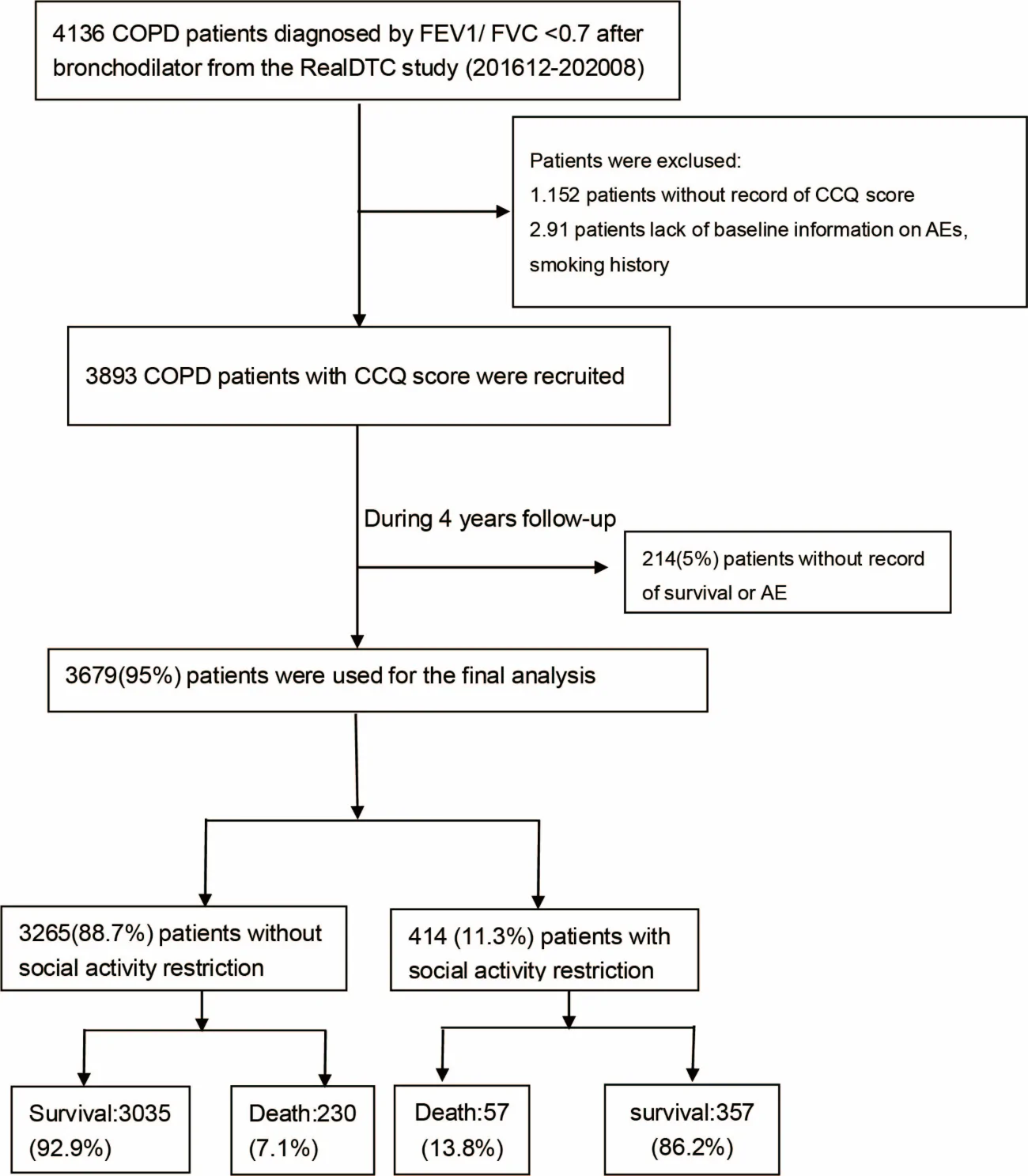
Flow diagram of the study.

**Table 1:** Baseline characteristics and clinical outcomes of COPD patients with and without restricted social activity*

	Total	Without restricted social activity	With restricted social activity	*P*-value
**Total**	3,679 (100)	3,265 (88.7)	414 (11.3)	
**Age in years, x̄ (SD)**	65.72 (9.1)	65.45 (9.1)	67.81 (8.4)	<0.001
<65	1,494 (40.6)	1,360 (41.7)	134 (32.4)	<0.001
≥65	2,185 (59.4)	1,905 (58.3)	280 (67.6)	
**Gender**				0.353
Male	3,114 (84.6)	2,770 (84.8)	344 (83.1)	
Female	565 (15.4)	495 (15.2)	70 (16.9)	
**Marital status**				0.004
Married status†	3,479 (94.6)	3,102 (95.0)	377 (91.1)	
Unmarried status	42 (1.1)	35 (1.1)	7 (1.7)	
Widowhood†	158 (4.3)	128 (3.9)	30 (7.2)	
**Education**				0.287
Junior high school and below	1,511 (41.1)	1,351 (41.4)	160 (38.6)	
High school and above	2,168 (58.9)	1,914 (58.6)	254 (61.4)	
**Smoking status**				0.352
Never smoker	705 (19.5)	622 (19.4)	83 (20.4)	
Ex-smoker	1,333 (36.9)	1,173 (36.6)	160 (39.3)	
Current smoker	1,577 (43.6)	1,413 (44.0)	164 (40.3)	
**BMI, kg/m^2^, x̄ (SD)**	22.58 (3.4)	22.63 (3.3)	22.15 (3.6)	0.007
**FEV_1_(L), x̄ (SD)**	1.29 (0.5)	1.34 (0.5)	1.03 (0.4)	<0.001
**FEV_1_%pred, x̄ (SD)**	52.50 (19.9)	53.72 (20.0)	43.68 (17.1)	<0.001
**FEV_1_/FVC, x̄ (SD)**	47.07 (12.0)	47.69 (12.0)	42.61 (11.6)	<0.001
**GOLD grades**				<0.001
1–2	1,992 (54.1)	1852 (56.7)	140 (33.8)	
3–4	1,687 (45.9)	1,413 (43.3)	274 (66.2)	
**GOLD group**				<0.001
A†	654 (17.8)	631 (19.3)	23 (5.6)	
B	1,420 (38.6)	1,259 (38.6)	161 (38.9)	
C†	228 (6.2)	221 (6.8)	7 (1.7)	
D†	1,377 (37.4)	1,154 (35.3)	223 (53.9)	
**Exacerbation in the past year**				<0.001
No	1,681 (45.7)	1,545 (47.3)	136 (32.9)	
Yes	1,998 (54.3)	1,720 (52.7)	278 (67.1)	
**CAT score, x̄ (SD)**	14.89 (6.7)	14.28 (6.5)	19.71 (7.1)	<0.001
<10	883 (24.0)	853 (26.1)	30 (7.2)	
≥10	2,786 (76.0)	2,412 (73.9)	384 (92.8)	
**mMRC score, x̄ (SD)**	2.00 (1.0)	1.92 (1.0)	2.64 (1.0)	<0.001
0–1	1,141 (31.0)	1,084 (33.2)	57 (13.8)	<0.001
2–4	2,538 (69.0)	2,181 (66.8)	357 (86.2)	
CCQ, x̄ (SD)	21.49 (6.4)	20.52 (6.4	29.12 (6.6)	<0.001
**COVID-19 pandemic**				<0.001
Pre-pandemic	3,083 (83.8)	2,778 (90.1)	305 (73.2)	
Post-pandemic	596 (16.2)	487 (9.9)	109 (26.3)	
**Exacerbation in the first year**				<0.001
No	2,502 (68.0)	2,253 (69.0)	249 (60.1)	
Yes	1,177 (32.0)	1,012 (31.0)	165 (39.9)	
**Sever exacerbation in the first year**				0.001
No	2,964 (80.6)	2,656 (81.3)	308 (74.4)	
Yes	715 (19.4)	609 (18.7)	106 (25.6)	
**Mortality in the first year**				<0.001
No	3,590 (97.6)	3,201 (98.0)	389 (94.0)	
Yes	89 (2.4)	64 (2.0)	25 (4.0)	
**Mortality during four-year follow-up period**				<0.001
No	3,392 (92.2)	3,035 (93.0)	357 (86.2)	
Yes	287 (0.8)	230 (7.0)	57 (13.8)	

CAT – COPD assessment test, COPD – chronic obstructive pulmonary disease, FEV_1_ – forced expiratory volume in 1 s, FVC – forced vital capacity, GOLD – Global Initiative for Chronic Obstructive Lung Disease, mMRC – modified Medical Research Council dyspnea scale, SD – standard deviation, x̄ – mean

*Presented as n (%) unless specified otherwise.

†Considered statistically significant.

### Factors associated with restricted social activity in COPD patients

Binary logistic regression analysis identified several factors positively associated with restricted social activity. These included older age (adjusted odds ratio (aOR) = 1.389, *P* = 0.005), widowhood (aOR = 1.760, *P* = 0.011), more severe disease (GOLD grades 3–4: aOR = 2.121; GOLD group B: aOR = 2.859; GOLD group D: aOR = 3.927; all *P* < 0.001), a history of acute exacerbations (aOR = 1.478, *P* = 0.001), higher symptom burden (CAT score of ≥10: aOR = 2.760; mMRC score of ≥2: aOR = 1.782; CCQ: aOR = 1.250; all *P* < 0.001), and enrolment during the post-pandemic period (aOR = 2.654, *P* < 0.001). Conversely, better lung function was protective. Higher FEV_1_ (aOR = 0.317), FEV_1_%pred (aOR = 0.975), and FEV_1_/FVC ratio (aOR = 0.970) were all negatively associated with restricted social activity (all *P* < 0.001) (**Table 2**).

**Table 2:** Multivariate analysis of independent relative factors for COPD patients with restricted social activity, adjusted for age, BMI, pulmonary function, GOLD grades, GOLD group, exacerbation in the past year, CAT, CCQ, and mMRC

	aOR (95% CI)	*P*-value
**Age in years**		
<65	ref	
≥65	1.389 (1.103–1.749)	0.005
**Marital status**		
Married status	ref	
Unmarried status	1.725 (0.744–4.00)	0.204
Widowhood	1.760 (1.139–2.718)	0.011
**BMI, kg/m^2^**	0.992 (0.962–1.023)	0.619
**FEV_1_(L)**	0.317 (0.239–0.420)	<0.001
**FEV_1_%pred**	0.975 (0.968–0.981)	<0.001
**FEV_1_/FVC**	0.970 (0.960–0.980)	<0.001
**GOLD grades**		
1–2	ref	
3–4	2.121 (1.688–2.666)	<0.001
**GOLD group**		
A	ref	
B	2.859 (1.815–4.504)	<0.001
C	0.770 (0.324–1.895)	0.553
D	3.927 (2.501–6.167)	<0.001
**Exacerbation in the past year**		
No	ref	
Yes	1.478 (1.169–1.869)	0.001
**CAT score**		
<10	ref	
≥10	2.760 (1.860–4.093)	<0.001
**mMRC score**		
0–1	ref	
2–4	1.782 (1.298–2.477)	<0.001
CCQ	1.250 (1.221–1.280)	<0.001
**COVID-19 pandemic**		
Pre-pandemic	ref	
Post-pandemic	2.654 (2.059-3.421)	<0.001

aOR – adjusted odds ratio, CAT – COPD assessment test, CI – confidence interval, COPD – chronic obstructive pulmonary disease, FEV_1_ – forced expiratory volume in 1 s, FVC – forced vital capacity, GOLD – Global Initiative for Chronic Obstructive Lung Disease, mMRC – modified Medical Research Council dyspnea scale, ref – reference

### Future acute exacerbation and mortality among COPD patients with restricted social activity

Compared with patients without restricted social activity, those with restricted social activity had higher rates of acute exacerbation (39.9% *vs.* 31.0%, *P* < 0.001), severe acute exacerbation (25.6% *vs.* 18.7%, *P* = 0.001), and mortality (4.0% *vs.* 2.0%, *P* < 0.001) during the one-year follow-up period (**Table 1**). In multivariable analyses, patients with restricted social activity had a higher risk of acute exacerbation (aOR = 1.320; 95% confidence interval (CI) = 1.100–1.583, *P* = 0.003), severe acute exacerbation (aOR = 1.299; 95% = 1.014–1.664, *P* = 0.038), and mortality (aOR = 2.683; 95% CI = 1.646–4.374, *P* < 0.001) (Table S2 in the **Online Supplementary Document**). During the four-year follow-up period, mortality remained higher in the socially restricted group than in the non-restricted group (13.8% *vs.* 7.0%, *P* < 0.001). Kaplan-Meier analysis showed that patients with restricted social activity had a higher cumulative risk of mortality than those without restricted social activity (hazard ratio (HR) = 1.752; 95% CI = 1.298–2.364, *P* < 0.001) (**Figure 2**). Furthermore, compared with patients without restricted social activity, mortality risk was higher among those with social activity restriction scores of 2–3 (HR = 1.601; 95% CI = 1.134–2.261, *P* < 0.001) and 4–6 (HR = 4.767; 95% CI = 2.485–9.142, *P* < 0.001) (**Figure 3**).

**Figure 2:**
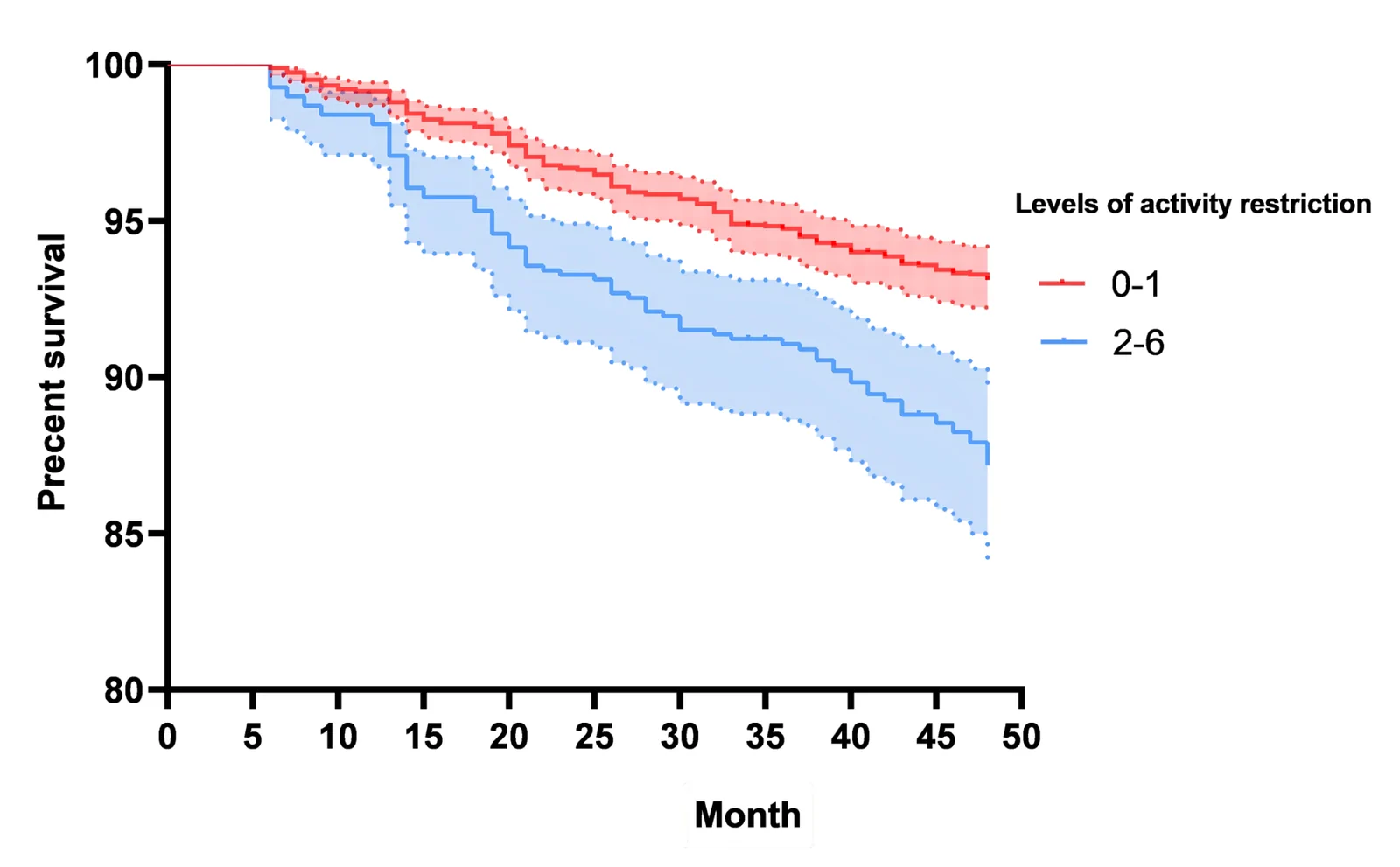
Risk of all-cause mortality in chronic obstructive pulmonary disease patients with restricted social activity. Log-rank *P* < 0.001, HR_2-6_ = 1.752 (95% CI = 1.298–2.364). CI – confidence interval, HR – hazard ratio.

**Figure 3:**
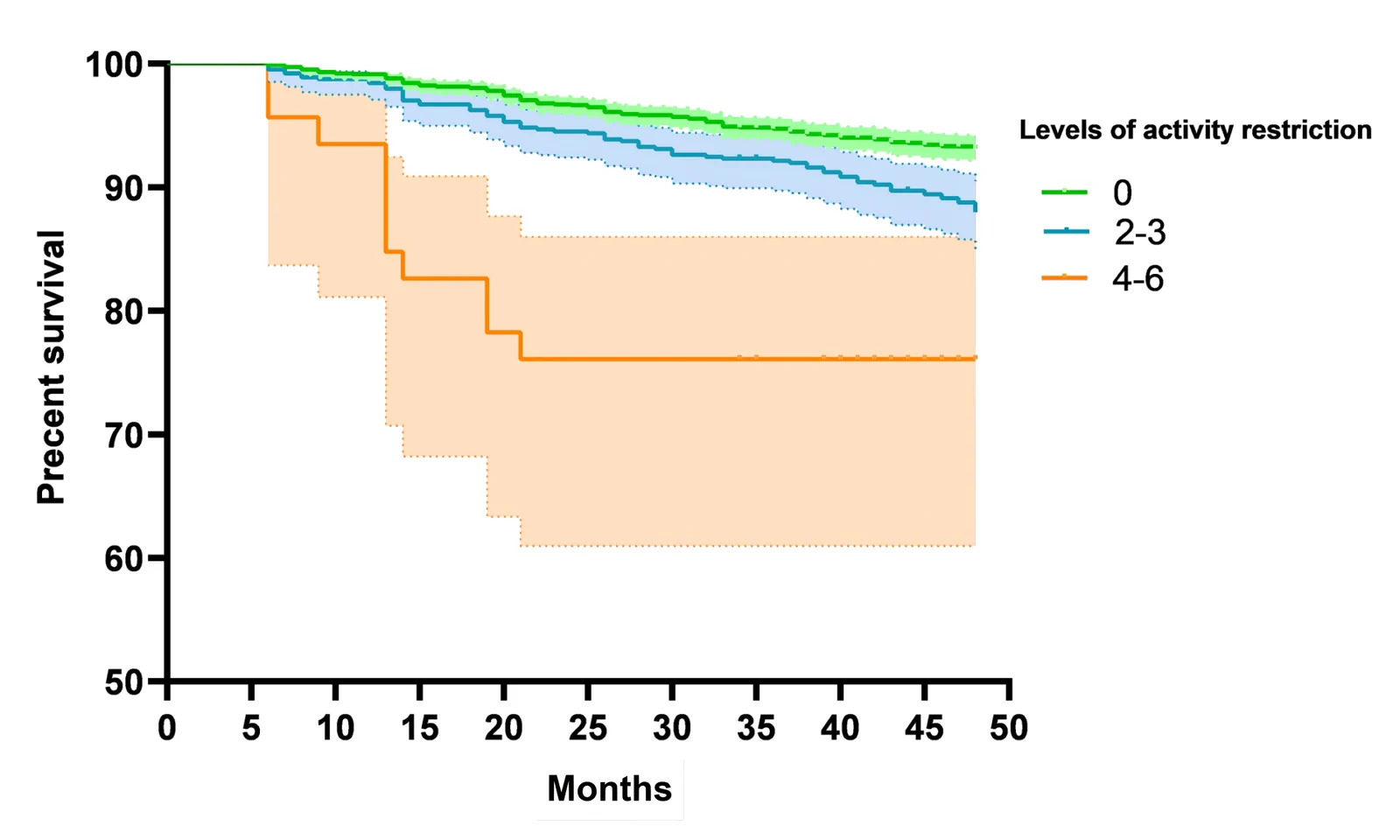
Risk of all-cause mortality in chronic obstructive pulmonary disease patients with different levels of restricted social activity. Log-rank *P* < 0.05, HR_2-3_ = 1.601 (95% CI = 1.134–2.261), HR_4-6_ = 4.767 (95% CI = 2.485–9.142). CI – confidence interval, HR – hazard ratio.

### Risk factors associated with mortality during the four-year follow-up period according to Cox regression analysis

Cox regression analysis demonstrated that restricted social activity was an independent risk factor for mortality (HR = 1.752; 95% CI = 1.298–2.364, *P* < 0.001). In the sensitivity analysis excluding widowed patients, social activity restriction remained independently associated with mortality (HR = 1.794; 95% CI = 1.318–2.441, *P* < 0.001) (Table S4 in the **Online Supplementary Document**), with effect estimates similar to those in the full cohort. Compared with patients whose social activity restriction scores were 0–1, those with scores of 2–3 (HR = 1.601; 95% CI = 1.134–2.261, *P* = 0.008) and 4–6 (HR = 4.767; 95% CI = 2.485–9.142, *P* < 0.001) had a higher risk of mortality. In addition, age of ≥65 years (HR = 2.245; 95% CI = 1.642–3.061, *P* < 0.001), GOLD group B (HR = 2.010, 95% CI = 1.191–3.391, *P* = 0.009), GOLD group D (HR = 2.252; 95% CI = 1.337–3.791, *P* = 0.002), a history of acute exacerbation in the previous year (HR = 1.348; 95% CI = 1.061–1.712, *P* = 0.015), CAT score of ≥10 (HR = 1.691; 95% CI = 1.156–2.245, *P* = 0.007), and mMRC score of ≥2 (HR = 1.737; 95% CI = 1.233–2.446, *P* = 0.002) were positively associated with mortality risk. Female sex (HR = 0.435; 95% CI = 0.249–0.759, *P* = 0.003), a high school education or above (HR = 0.644; 95% CI = 0.502–0.826, *P* = 0.001), and higher body mass index (HR = 0.920; 95% CI = 0.887–0.953, *P* < 0.001) were negatively associated with mortality risk (**Table 3**).

**Table 3:** Cox regression analysis of risk factors for all-cause mortality in patients with COPD, adjusted for age, gender, marital status, education, pulmonary function, GOLD group, exacerbation in the past year, CAT, mMRC, and restriction of social activity

	HR (95% CI)	*P*-value
**Age in years**		
<65	ref	
≥65	2.245 (1.642–3.061)	<0.001
**Gende**r		
Male	ref	
Female	0.435 (0.249–0.759)	0.003
**Marital status**		
Married status	ref	
Unmarried status	0.581 (0.143–2.353)	0.447
Widowhood	1.135 (0.697–1.849)	0.611
**Education**		
Junior high school and below	ref	
High school and above	0.644 (0.502–0.826)	0.001
**BMI, kg/m2**	0.920 (0.887–0.953)	<0.001
**GOLD group**		
A	ref	
B	2.010 (1.191–3.391)	0.009
C	1.581 (0.736–3.396)	0.241
D	2.252 (1.337–3.791)	0.002
**Exacerbation in the past year**		
No	ref	
Yes	1.348 (1.061–1.712)	0.015
**CAT**		
<10	ref	
≥10	1.691 (1.156–2.245)	0.007
**mMRC**		
0–1	ref	
2–4	1.737 (1.233–2.446)	0.002
**Restriction of social activity**		
No	ref	
Yes	1.752 (1.298–2.364)	<0.001
Level of restricted social activity*		
0–1	ref	
2–3	1.601 (1.134–2.261)	0.008
4–6	4.767 (2.485–9.1429	<0.001

CAT – COPD assessment test, CI – confidence interval, COPD – chronic obstructive pulmonary disease, FEV_1_ – forced expiratory volume in 1 s, FVC – forced vital capacity, GOLD – Global Initiative for Chronic Obstructive Lung Disease, HR – hazard ratio, mMRC – modified Medical Research Council dyspnea scale, ref – reference

*Adjusted for age, gender, marital status, education, smoking status, pulmonary function, GOLD group, exacerbation in the past year, CAT, and mMRC.

## DISCUSSION

This real-world prospective observational study yielded the following findings. First, 11.3% of outpatients with COPD had restricted social activities. Second, age, widowed status, FEV_1_, FEV_1_%pred, FEV_1_/FVC, GOLD grades 3–4, GOLD groups B and D, history of acute exacerbation, CAT score of ≥10, and mMRC score of ≥2 were independently associated with restricted social activity. Third, after multivariable adjustment, restricted social activity was an independent risk factor for future acute exacerbation and mortality in patients with COPD. Finally, the greater the degree of social activity restriction, the higher the risk of mortality.

Previous studies have reported that restricted social activity is not uncommon among patients with COPD. In a nationally representative cross-sectional study, Suen *et al.* reported that restricted social activity was present in approximately one in six patients with COPD [19]. In our study, the prevalence of restricted social activity was 11.3%, which was lower than that reported in Suen *et al.*’s study [19].

This difference may be because we assessed social activity restriction using a single item from the CCQ rather than a dedicated social isolation or social network instrument.

Social activity restriction in patients with COPD is likely multifactorial. Most importantly, disease-related factors, including dyspnoea, exercise intolerance, fatigue, and comorbidities, may directly reduce patients’ willingness or ability to participate in social activities [20]. The results of our study suggest that patients with restricted social activities were older, had a greater symptom burden, and had poorer lung function. Similar to the findings reported by Kochovska *et al.* [13] and Evangelista *et al.* [21], we observed a strong association between restricted social activity, poorer lung function, and more severe dyspnoea. Meanwhile, older patients with COPD tend to have more severe symptoms and a higher burden of comorbidities, making them more likely to experience restricted social activities [22,23]. In addition, healthcare-related factors may also play a role, including conservative medical advice, limited access to structured pulmonary rehabilitation, insufficient community-based COPD management, and a lack of individualised guidance regarding safe physical and social activity [24]. Moreover, the COVID-19 pandemic is an important contextual factor that may have contributed to social activity restriction in part of our cohort. During the pandemic, public health measures, such as lockdowns, home isolation, quarantine, travel restrictions, and physical distancing, substantially limited social participation. Because patients with COPD were considered at increased risk of severe respiratory infection, social activities may have been further reduced because of policy restrictions, medical advice, or fear of infection. Our study showed that patients with COPD enrolled after the onset of the COVID-19 pandemic had a higher prevalence of restricted social activity. Therefore, the social activity restriction may reflect both disease-related limitations and pandemic-related external restrictions, especially among patients assessed in 2020 and thereafter.

Acute exacerbation is another important adverse event in the course of COPD [25]. In our study, patients with restricted social activities had higher rates of future acute exacerbation and hospitalisation. These patients also had more severe symptoms, poorer lung function, and more acute exacerbations in the previous year, all of which may increase the likelihood of future exacerbations. After adjustment for age, symptoms, acute exacerbations in the previous year, and lung function, restricted social activity remained an independent risk factor for acute exacerbation and hospitalisation. Jordan *et al.* [26] and Bu *et al.* [27] found that social disengagement was associated with a significantly increased risk of hospital admission for respiratory diseases in the general population. Similarly, Iyer *et al.* [15] observed that limited movement space was associated with an increased risk of severe exacerbation in patients with COPD.

We found that restricted social activity was an independent risk factor for both short- and long-term mortality in patients with COPD. A meta-analysis of 90 cohort studies found that reduced social activity was associated with an increased risk of mortality in the general population [28]. Aartsen *et al.* [29] reported that reduced social activity increased the risk of mortality over 20 years by 16% in women and 15% in men in Norway. Suen *et al.* [30] followed 1241 patients with COPD for 4.4 years and found that reduced social activity was associated with a 1.35-fold increased risk of mortality. However, Suen *et al.* did not adjust for COPD severity [30], whereas a strength of our study was the inclusion of disease severity measures in the multivariable analyses. Several interrelated mechanisms may explain how restricted social activity contributes to worse COPD outcomes, and a central pathway involves physical deconditioning. Restricted social activity is often accompanied by reduced physical activity, which may contribute to peripheral skeletal muscle dysfunction in COPD, including muscle atrophy, fibre-type shifts toward a less oxidative phenotype, and impaired oxidative capacity [31]. These changes can reduce exercise tolerance and increase ventilatory requirements during exertion, thereby aggravating exertional dyspnoea and dynamic hyperinflation [32]. This may further discourage activity, creating a self-perpetuating dyspnoea–inactivity–deconditioning cycle that promotes frailty and sarcopenia, both of which are predictors of exacerbation and mortality [6]. In parallel, social restriction has been associated with depression, anxiety, and loneliness [33], which may heighten symptom perception, impair treatment adherence and participation in rehabilitation, and contribute to systemic inflammation and neuroendocrine dysregulation [8,34,35]. Given the observational design of our study, these pathways should be regarded as plausible and potentially interacting mechanisms rather than confirmed causal links.

In summary, our findings may have important clinical implications. Social prescribing, in which patients are referred to community-based resources such as peer-support networks, community groups, and structured social activities, may help reduce loneliness and improve well-being and may be particularly relevant for the socially restricted, older, and widowed patients [36]. Pulmonary rehabilitation programmes that integrate psychosocial support, mental health screening, and group-based activities could simultaneously address physical deconditioning and social isolation, potentially interrupting the dyspnoea–inactivity–deconditioning cycle [37]. Home-based or telerehabilitation models that incorporate remote psychosocial support may also help maintain engagement when in-person attendance is limited. Routine screening for social activity restriction using simple assessment tools could help identify high-risk patients who may benefit from targeted referral to these interventions. This issue may be especially relevant during epidemics or pandemics, when public health measures such as lockdowns, home isolation, travel restrictions, and physical distancing can substantially reduce social participation among older adults and patients with chronic respiratory disease [38,39]. As our study was observational, these potential interventions warrant evaluation in future prospective and interventional studies.

### Limitations

Our study has several limitations. First, the proportion of male patients was very high (84.6%). This male predominance is consistent with the epidemiology of COPD in China [3], where the disease occurs predominantly in men because of the substantially higher prevalence of cigarette smoking among men than among women. Although this sex distribution represents the Chinese COPD population, it limits the generalisability of our findings to female patients. Therefore, our findings should be applied to women with caution, and future studies with greater representation of female patients or dedicated sex-stratified analyses are warranted to determine whether the prognostic value of social activity restriction is consistent across sexes. Second, we assessed social activity restriction using a single CCQ domain rather than a dedicated social isolation or social network instrument. Although this item reflects disease-related social participation, it does not fully capture the broader constructs of social isolation and loneliness. Future studies should examine the correlation between CCQ-based measures and established social isolation instruments, such as the Lubben Social Network Scale or the UCLA Loneliness Scale, to further validate this approach.

## CONCLUSIONS

Restricted social activity was not uncommon among patients with COPD. Social activity restriction should be routinely assessed in COPD clinics as a simple marker for identifying patients at increased risk of exacerbation and mortality. During future epidemics or pandemics, public health responses for individuals with chronic respiratory disease should be more flexible, risk-stratified, and patient-centred. Future interventional and policy studies are needed to determine whether systematic assessment of social activity restriction and targeted supportive programs can improve outcomes in socially restricted patients with COPD.

## Additional material


Online Supplementary Document

## Data Availability

**Data availability:** All data of this study are available from the corresponding author, Ping Chen, upon reasonable request.
