# Supplementary material for: Associated factors and outcomes of social activity restriction in patients with chronic obstructive pulmonary disease: a real-world longitudinal cohort study
Source: J Glob Health. 2026 Oct 23;16:04295. doi: 10.7189/jogh.16.04295 (PMC13693292; doi:10.7189/jogh.16.04295)
Supplement: Supplementary file 1 [file jogh-16-04295-s001.pdf]

Supplement to: Liu C, Zhou Z, Song Q, Li T, Zhang P, Lin L, Peng Y, Zeng Y, ChenY, Cai S, Chen P. Associated factors and outcomes of social activity restriction in patientswith chronic obstructive pulmonary disease: a real-world longitudinal cohort study. J GlobHealth. 2026;16:04295.

Supplementary Table 1. Baseline characteristics of COPD patients.

	Total
Total	3679(100%)
Age, years	65.72±9.1
40-49	135(3.6%)
50-59	831(22.6%)
60-69	1407(38.3%)
70-79	1109(30.1%)
80-89	187(5.1%)
≥90	10(0.3%)
Gender	
Male	3114(84.6%)
Female	565(15.4%)
Marital status	
Married status	3479(94.6%)
Unmarried status	42(1.1%)
Widowhood	158(4.3%)
Education	
Junior high school and below	1511(41.1%)
High school and above	2168(58.9%)
Smoking status	
Never smoker	705(19.5%)
Ex-smoker	1333(36.9%)
Current smoker	1577(43.6%)
BMI, kg/m^2	22.58±3.4
FEV1(L)	1.29±0.5
FEV1%pred	52.50±19.9
FEV1/FVC	47.07±12.0
GOLD grades	
1 to 2	1992(54.1%)
3 to 4	1687(45.9%)
GOLD group	
A	654(17.8%)
B	1420(38.6%)
C	228(6.2%)
D	1377(37.4%)
Exacerbation in the past year	
No	1681(45.7%)

Yes	1998(54.3%)
CAT score	14.89±6.7
<10	883(24.0%)
≥10	2786(76.0%)
mMRC score	2.00±1.0
0-1	1141(31.0%)
2-4	2538(69.0%)
CCQ	21.49±6.4

Abbreviations: CAT, COPD assessment test; COPD, chronic obstructive pulmonary disease; FEV1, forced expiratory volume in 1 second; FVC, forced vital capacity; GOLD, global initiative for chronic obstructive lung disease; mMRC, modified medical research council dyspnea Scale.

Supplementary Table 2. Multivariate analysis of relative factors for exacerbation and mortality during one year follow-up of COPD

Variable	Acute exacerbation	*P* value	Sever exacerbation	*P* value	Mortality	*P* value
Restriction of social activity						
No	Reference		Reference		Reference	
Yes	1.320(1.100-1.583)	0.003	1.299(1.014-1.664)	0.038	2.683(1.646-4.374)	<0.001

Note: Adjusted for age, gender, marital status, education level, smoking status, exacerbation in the past year, CAT, pulmonary function.

Abbreviations: CAT, COPD assessment test; COPD, chronic obstructive pulmonary disease; GOLD, global Initiative for chronic obstructive lung disease; mMRC, modified medical research council dyspnea Scale; aOR, adjusted Odds Ratio; 95% CI, 95% confidence interval.

Supplementary Table 3. Clinical characteristics of the deceased COPD patients at baseline

	Survival, n (%)	Death, n (%)	*P* value
Total	3392(92.2%)	287(7.8%)	
Age, years	65.29±9.0	70.79±7.7	<0.001
<65	1440(42.5%)	54(18.8%)	<0.001
≥65	1952(57.5%)	233(81.2%)	
Gender			0.016
Male	2857(84.2%)	257(89.5%)	
Female	535(15.8%)	30(10.5%)	
Marital status			0.178
Married status	3212(94.7%)	267(93.0%)	
Unmarried status	40(1.2%)	2(0.7%)	
Widowhood	140(4.1%)	18(6.3%)	
Education			<0.001
Junior high school and below	1349(39.8%)	162(56.4%)	
High school and above	2043(60.2%)	125(43.6%)	
Smoking status			0.130
Never smoker	658(19.8%)	47(16.5%)	
Ex-smoker	1213(36.4%)	120(42.1%)	
Current smoker	1459(43.8%)	118(41.4%)	
Occupational exposure			0.955
No	2098(61.9%)	178(62.0%)	
Yes	1294(38.1%)	109(38.0%)	
Biofuel exposure			<0.001
No	2137(63.0%)	144(50.2%)	
Yes	1255(37.0%)	143(49.8%)	
BMI, kg/m^2	22.69±3.4	21.31±3.4	<0.001
FEV1(L)	1.31±0.5	1.13±0.5	<0.001
FEV1%pred	52.67±19.7	50.61±22.7	0.116
FEV1/FVC	47.12±11.9	46.47±13.1	0.411
GOLD grades			0.002
1 to 2	1862(54.9%)	130(45.3%)	
3 to 4	1530(45.1%)	157(54.7%)	
GOLD group			<0.001
A*	6358(18.7%)	19(6.6%)	
B	1311(38.6%)	109(38.0%)	
C	216(6.4%)	12(4.2%)	
D*	1230(36.3%)	147(51.2%)	

Exacerbation in the past year			0.002
No	1575(46.4%)	106(36.9%)	
Yes	1817(53.6%)	181(63.1%)	
CAT score	14.60±6.7	18.39±6.9	<0.001
<10	851(25.1%)	32(11.1%)	<0.001
≥10	2541(74.9%)	255(88.9%)	
mMRC score	1.96±1.0	2.54±0.9	<0.001
0-1	1098(32.4%)	43(15.0%)	<0.001
2-4	2294(67.6%)	244(85.0%)	
CCQ	21.23±6.9	24.66±7.2	<0.001
Restriction of social activity			<0.001
No	3035(89.5%)	230(80.1%)	
Yes	357(10.5%)	57(19.9%)	
Level of restricted social activity			<0.001
0-1*	3035(89.5%)	230(80.1%)	
2-3*	323(9.5%)	44(15.3%)	
4-6*	34(1.0%)	13(4.5%)	

Note: Adjusted for age, BMI, pulmonary function, GOLD grades, GOLD group, exacerbation in the past year, CAT, CCQ, mMRC. *: It was considered statistically significant.

Abbreviations: CAT, COPD assessment test; CCQ, clinical COPD questionaire; COPD, chronic obstructive pulmonary disease; GOLD, global Initiative for chronic obstructive lung disease; mMRC, modified medical research council dyspnea Scale.

Supplementary Table 4. Multivariate analysis of risk factors for all-cause mortality in patients with COPD after excluding widowed patients

Variable	aOR	95% CI	*P value*
Age, years			
<65	Reference		
≥65	2.301	1.663-3.185	<0.001
Gender			
Male	Reference		
Female	0.403	0.220-0.740	0.003
Education			
Junior high school and below	Reference		
High school and above	0.648	0.501-0.839	0.001
BMI, kg/m2	0.925	0.892-0.960	<0.001
GOLD group			
A	Reference		
B	2.010	1.191-3.391	0.009
C	1.581	0.736-3.396	0.241
D	2.252	1.337-3.791	0.002
Exacerbation in the past year			
No	Reference		
Yes	1.348	1.061-1.712	0.015
CAT			
<10	Reference		
≥10	1.766	1.187-2.267	0.005
mMRC			
0-1	Reference		
2-4	1.73	1.233-2.446	0.002
Restriction of social activity			
No	Reference		
Yes	1.794	1.318-2.441	<0.001
Level of restricted social activity*			
0-1	Reference		
2-3	1.528	1.085-2.151	0.015
4-6	4.284	2.377-7.719	<0.001

Note: Adjusted for age, gender, education, pulmonary function, GOLD group, exacerbation in the past year, CAT, mMRC, restriction of social activity. *: Adjusted for age, gender, education, smoking status, pulmonary function, GOLD group, exacerbation in the past year, CAT, mMRC.

Abbreviations: CAT, COPD assessment test; COPD, chronic obstructive pulmonary disease; GOLD, global Initiative for chronic obstructive lung disease; mMRC, modified medical research council

dyspnea Scale; HR, Hazard Ratio; 95% CI, 95% confidence interval.
